# Protective Effect of Anthocyanins Extract from Blueberry on TNBS-Induced IBD Model of Mice

**DOI:** 10.1093/ecam/neq040

**Published:** 2011-04-14

**Authors:** Lin-Hua Wu, Zeng-Lai Xu, Di Dong, Shan-An He, Hong Yu

**Affiliations:** ^1^Department of Pharmacy, The Second Clinical College of Harbin Medical University, Harbin, China; ^2^Institute of Botany, Jiangsu Province & Chinese Academy of Sciences, Jiangsu Province Key Laboratory for Plant Ex-Situ Conservation, Nanjing 210014, China

## Abstract

This study was carried out to evaluate the protective effect of anthocyanins extract of blueberry on trinitrobenzene sulfonic acid (TNBS)-induced inflammatory bowel disease (IBD) model of mice. The study employed female C57BL/6 mice (*n* = 50), and colitis was induced by intracolonic injection of 0.5 mg of TNBS dissolved in 50% ethanol–phosphate buffered solution. The mice were divided into five groups (*n* = 10): vehicle, TNBS control and anthocyanins groups that received different doses of anthocyanins extract (10, 20 and 40 mg kg^−1^) daily for 6 days. Both increase in body weight and diarrhea symptoms were monitored each day. After 6 days, the animals were killed, and the following parameters were assessed: colon length, morphological score, histological score and biochemical assay (NO, myeloperoxidase (MPO), interleukin (IL)-12, IL-10, tumor necrosis factor (TNF)-**α** and interferon (IFN)-**γ**). The results showed that the anthocyanins extract of blueberry rendered strong protection against TNBS-induced colonic damage at a dosage of 40 mg kg^−1^. When compared with the control, anthocyanins extract significantly prevented loss of body weight and ameliorated the scores of diarrhea, morphology and histology. Treatment with anthocyanins extract restored IL-10 excretion, as well as caused reduction in the levels of NO, MPO, IL-12, TNF-**α** and IFN-**γ**. Our research revealed the protective effect of anthocyanins extract from blueberry on TNBS-induced experimental colitis in mice, as well as examined whether high levels of dietary blueberries would lower the risk or have protective effects on human IBD, which may require further investigation.

## 1. Introduction

**Figure 1:**
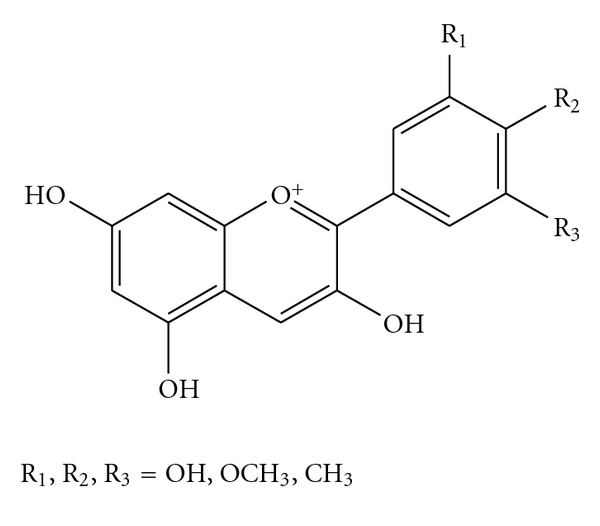
General structure of anthocyanidins (aglycons).

Blueberries are among the fruits that are best recognized for their potential health benefits [1], and many of the heath-promoting properties of blueberries are thought to be attributable to anthocyanins that structurally belong to the natural products of flavonoids (Figure 1). Anthocyanins are water-soluble pigments that might appear as red, purple or blue pigments according to their pH levels and are present in blueberries at high concentrations [2]. Anthocyanins from blueberries are also used as anti-inflammatory, antimutagenic and rhodophylactic agents, and the principal therapeutic benefits attributable to anthocyanins include antioxidant protection and maintenance of DNA integrity [3, 4].

Inflammatory bowel disease (IBD) is a chronic, relapsing, idiopathic inflammation of the gastrointestinal tract. Patients suffering from IBD experience a set of gastrointestinal symptoms, and their quality of life is also limited by some complications such as: toxic megacolon [5], bowel perforation [6] and surgical complications [7]. It often leads to long-term and sometimes irreversible impairment of gastrointestinal structure and function. Besides deteriorated living quality, IBD patients also have higher risk of colon cancer [8]. IBD has exhibited high prevalence in developed Western countries; however, in Asia, its prevalence is much lower [9]. Some believe that Oriental diet enriched with different kinds of flavonoids might be one of the most important factors rendering resistance to colon disease. Furthermore, some studies have revealed that anthocyanins have protective effect against colon cancer in rats [10]. As various evidences have demonstrated that there is a close interconnection between colitis and colon cancer, it was worth investigating the protective effect of anthocyanins on colitis, based on these aspects.

In this study, we reconstructed the trinitrobenzene sulfonic acid (TNBS)-induced colitis model in mice and monitored for diarrhea, the resulting loss of body weight and animal death every day in the whole experimental course. Subsequently, we investigated the function of neutrophilic granulocyte using myeloperoxidase (MPO) assay; in addition, the severity of colitis was also assessed by both macroscopical and histological detection. Finally, we detected the influence of anthocyanins on the production of interleukin (IL)-10 and -12, NO, tumor necrosis factor (TNF)-*α* and interferon (IFN)-*γ* in the tissues. Thus, evaluation of the protective effect of anthocyanins extract of blueberry on IBD might shed light on drug discovery or alternative therapy for IBD treatment.

## 2. Methods

### 2.1. Animals and Grouping

Female C57BL/6 mice were purchased from Experimental Animal Center of Harbin Medical University. The animals, 8–12 weeks of age, weighing 18–22 g, were kept under specific pathogen-free environment. They were maintained in plastic cages with free access to pellet food and water, at 21 ± 2°C and kept on a 12-h light/dark cycle. Animal welfare and experimental procedures were carried out strictly in accordance with the guidance for care and use of laboratory animals (National Research Council of USA, 1996) and the related ethical regulations of our university. All efforts were made to minimize the animal's suffering and to reduce the number of animals used. The mice were randomly divided into five groups, each containing 10 mice: vehicle group received 100 *μ*L of 50% ethanol-phosphate buffered solution (PBS) by rectal needle; control group received a single rectum injection of 0.5 mg of TNBS only; and experimental IBD mice received anthocyanins extract at doses of 10, 20 and 40 mg kg^−1^. The anthocyanins groups were orally administered with different doses of anthocyanins dissolved in 100 *μ*L of physiological saline once every day, and the vehicle and control groups were also administered with equal volume of physiological saline daily.

### 2.2. Therapeutic Agents and Reagents

Anthocyanins extract preparation: ripe blueberries (*Vaccinium ashei*) picked from the field were freeze-dried and then powdered. The powder was kept at −70° until further use. The freeze-dried powder was extracted using methanol/water/acetic acid (85 : 15 : 0.5, v/v, MeOH/H_2_O/AcOH) thrice as previously reported, and the anthocyanins content of the extract was about 32%, which was observed using UV-Vis spectra analysis [11, 12]. Subsequently, the solution was filtered and concentrated in vacuum under 40°C. The residue was lyophilized and kept at −70°C; TNBS (Sigma, St Louis, MO, USA), Nitric Oxide Assay Kit (Beyotime, Shanghai, China), MPO Assay Kit (Beyotime, Shanghai, USA), TNF-*α*, IFN-*γ*, IL-10 and -12 and ELISA Kit (R&D system, MN, USA) were used for the study.

### 2.3. Induction of TNBS Colitis

Colitis was induced by a single intracolonic injection of 0.5 mg of TNBS dissolved in 50% ethanol-PBS solution into the descending colon. The volume of TNBS enema was kept at 100 *μ*L. The mice were placed under low dosage of ether anesthesia; later, a rubber catheter lubricated with cosmolin was inserted into the colon through anus. TNBS was injected only when the tip of the catheter was 4 cm inside from the anus. The animals were observed for 6 days, and afterwards, killed under ether anesthesia by cervical dislocation, for the assessment of various parameters.

### 2.4. Assessment of TNBS Colitis

The increase in body weight, diarrhea score and mortality rate of the animals was monitored daily. Diarrhea was scored as follows: 0, normal; 2, loose previous term stools; and 4, diarrhea. After 6 days, the mice were killed, and colons were collected and evaluated for colon length and macroscopic score. The score of macroscopy was given based on the following criteria: 0, normal; 1, erythema only; 2, erythema, slight edema and small erosions; 3, two or more bleeding ulcers and/or inflammation and/or moderate adhesions; and 4, severe ulceration and/or stenosis with dilations and/or severe adhesions. We also collected the sample of colon, and parts of them were stored at −70°C for protein extraction and were fixed in formalin for histological assay.

### 2.5. Histological Score of Colitis

Fixed tissue was stained with hematoxylin and eosin for subsequent histological examination. We gave the macroscopic score according to the following criteria: 0, no signs of inflammation; 1, low level of leukocyte infiltration; 2, moderate level of leukocyte infiltration; 3, high level of leukocyte infiltration, high vascular density and thickening of bowel wall; and 4, transmural infiltrations, loss of goblet cells, high vascular density and strong bowel wall thickening.

### 2.6. MPO Activity Measurement

Colonic tissues from all the surviving mice of each group were partitioned and stored immediately at −70°C until further use. In all the measurement of different indicators, such as MPO activity, NO release and ELISA for cytokines, the data indicated the average value of samples from every surviving mouse in each group. All the experiments were performed within 1 week of collection of tissues. The MPO activity was measured according to the method described in the user's guide of the kit. The tissues were homogenized in hexadecyltrimethylammonium bromide in 50 mM of potassium phosphate buffer. Aliquots were then added to *O*-dianisidine hydrochloride solution. The absorbance was read at 450 nm using a microplate reader. MPO was expressed in units per milligram of tissue, where 1 U corresponded to the activity required to degrade 1 mmol of hydrogen peroxide in 1 min at 24°C.

### 2.7. NO Release Measurement

The kit principle was based on Greiss reagent. The tissues were smashed after incubation in liquid nitrogen and suspended in suspending buffer (NaCl 0.1 mol L^−1^, Tris–HCl 0.01 mol L^−1^, pH 7.6, egtazic acid 1 mmol L^−1^, aprotinin 1 mg L^−1^ and PMSF 100 mg L^−1^). After centrifugation, 50 *μ*L of Griess reagent (equal volume of 1% sulfanilamide in HCl of 0.1 mol L^−1^ and 0.1% *N*-(-1-naphthyl-ethylenediamine dihydrochloride)) was added to 50 *μ*L of the suspending media. Nitrite concentration was determined by spectrophotometry (560 nm) from a standard curve (0–100 mmol L^−1^) derived from NaNO_2_ (Beyotime Biotechnology). The NO data were expressed as mean (nitrite) in *μ*mol mg^−1^ tissue.

### 2.8. ELISA for IL-12 and -10, TNF-*α* and IFN-*γ*

The levels of IL-12 and -10, TNF-*α* and IFN-*γ* were measured using ELISA kit [13]. Mice colonic tissues of each group were homogenized in PBS and the final concentrations were 10% (w/v). The plates were read at 490 nm right after the chromogenic reaction stopped.

### 2.9. Statistical analysis

The data were expressed as mean ± SEM. Repeated measures ANOVA test was used to analyze the differences in the body weight gain between the groups, and other parameter differences between the groups were initially analyzed using Student's *t*-test. The level of significance was set at *P* <  .05. All the analyses were performed using SPSS 12.0 software.

## 3. Results

### 3.1. Mortality and Animal Body Weight

**Figure 2:**
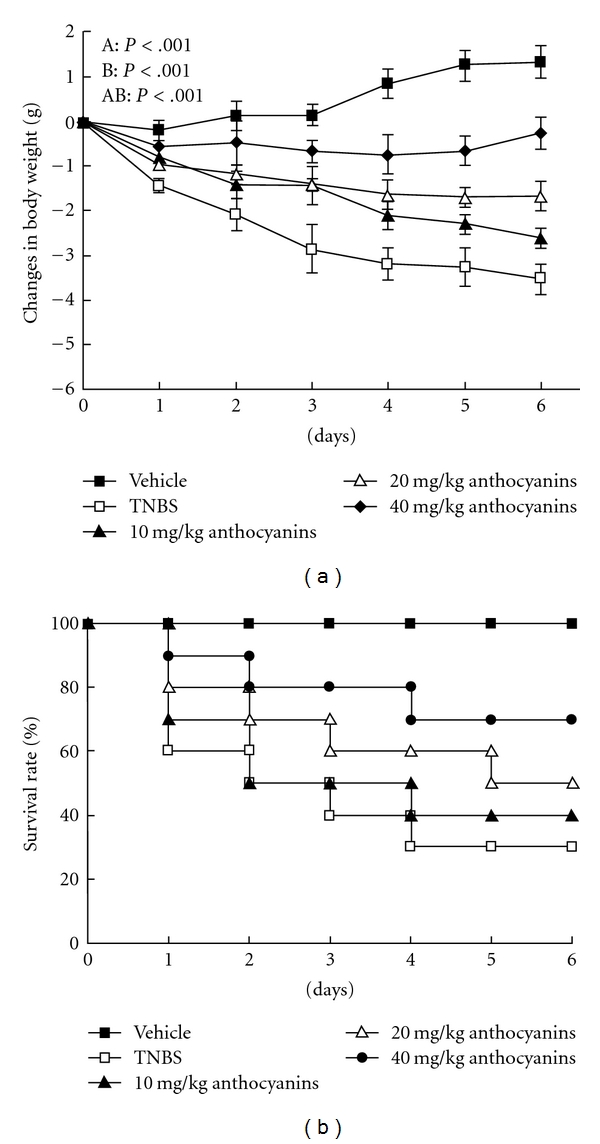
(a) Effects of anthocyanins extract on body weight in mice with TNBS-induced colitis. TNBS-induced colitis resulted in the loss of animal body weight, and the body weight varied during the administration of different doses (10, 20 and 40 mg kg^−1^) of anthocyanins, TNBS or vehicle in 6 days. The data were expressed as mean ± SEM. *P*-values were calculated using repeated measures ANOVA. A, differences between the groups; B, differences over time; and AB, differences between the interaction of experiment and time. Pairwise comparisons between the groups at individual time points were conducted by ANOVA and LSD *post hoc* test. On Days 1, 2 and 3, all pairwise comparisons between the groups had significant differences, except 10 and 20 mg kg^−1^ groups (*P* <  .001). On Days 4, 5 and 6, all pairwise comparisons between the groups had significant differences (*P* <  .001). (b) Effects of anthocyanins extract on the survival rate in mice with TNBS-induced colitis. TNBS-induced colitis resulted in animal death in the whole experiment course when compared with the TNBS-group mice that were given 20 and 40 mg kg^−1^ anthocyanins extract, which showed much less mortality rate.

The body weight gain of each mouse was determined every day by comparing the current weight with the weight on Day 0, and the animal mortality was also observed. As shown in Figure 2(a), when compared with the vehicle group, the body weight of the TNBS-treated mice decreased; however, when compared with the control, the body weight of mice administered with 40 mg kg^−1^ of anthocyanins extract had significant recovery. As shown in Figure 2(b), at the end of the experiment, the number of dead mice were 0 (0/10), 7 (7/10), 6 (6/10), 5 (5/10) and 3 (3/10) for vehicle, TNBS control, and mice treated with anthocyanins extract of 10, 20, and 40 mg kg^−1^, respectively. It is clear that the mortality rate of mice that were given high dosage of anthocyanins extract was lesser.

### 3.2. Diarrhea Scoring

**Figure 3:**
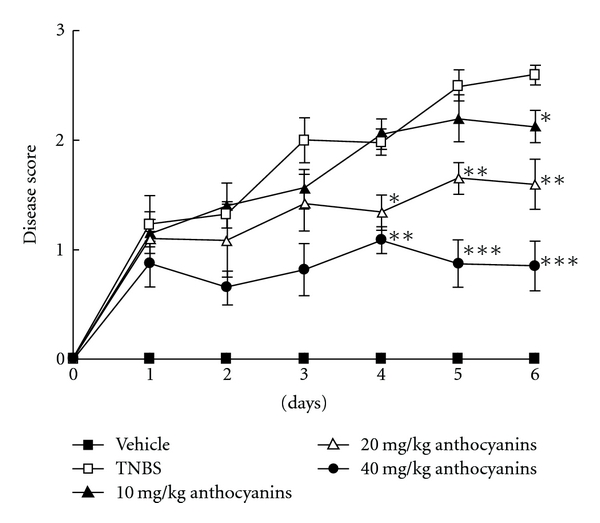
Effects of anthocyanins extract on mice diarrhea score in TNBS-induced colitis in mice. The main symptom of colitis was diarrhea; serious diarrhea symptoms occurred in TNBS group and did not cease until the experiment was terminated; administration of anthocyanins extract significantly reduced the diarrhea score in the whole experiment course. The scores were given according to the criteria presented in the method; *P < .05, **P < .01, ***P < .005 versus TNBS group.

During the entire experiment, mice stool consistency was monitored every day. As shown in Figure 3, simple TNBS injection significantly increased the diarrhea score of the control; however, all doses of anthocyanins extract were found to inhibit the score, which that increased in a dose-dependent manner (*P* <  .01). Furthermore, anthocyanins extract was observed to relieve the diarrhea symptom induced by TNBS.

### 3.3. Colon Length Change and Macroscopic Score

**Figure 4:**
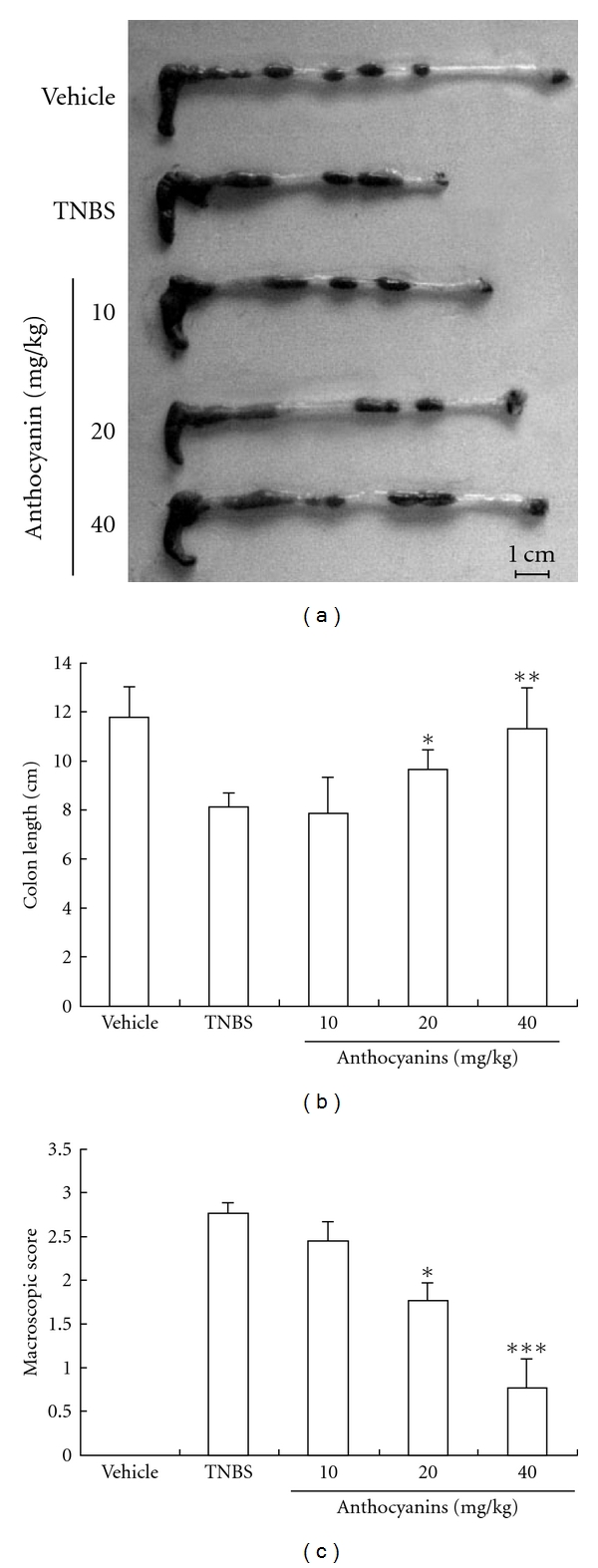
(a–c) Effects of anthocyanins extract on colon length and macroscopic score in mice with TNBS-induced colitis. The colon length of the surviving mice from each group was measured and typical sample was filmed, and the inflammatory severity of colon was scored. Both these indicators were ameliorated by anthocyanins extract. The scores were given according to the criteria presented in the method. *P < .05, **P < .01, ***P < .005 versus TNBS group.

Figure 4(a) presents an intuitionistic data of the protective effect of anthocyanins extract; the samples in the figure are the representative colons of each group. As shown in Figure 4(b), administration of high dose of anthocyanins extract significantly reversed the shortening of the colons owing to inflammation and hydropsia of mice colons induced by TNBS injection, and this result also had high coincidence with the macroscopic score presented in Figure 4(c).

### 3.4. Histological Analysis

**Figure 5:**
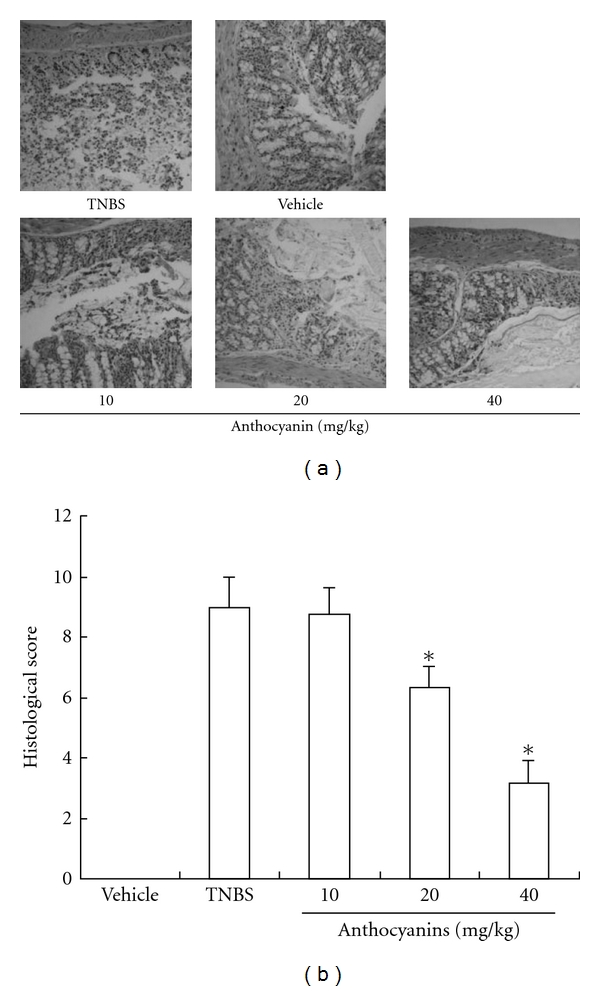
Results of anthocyanins extract on histological analysis in mice with TNBS-induced colitis. Slices were inspected by a certified pathologist, and the inflammatory severity of colonic tissues from survived mice in each group was scored and filmed. As shown (a and b), samples from mice that were given anthocyanins extract exhibited minor inflammatory reaction when compared with the TNBS group. The scores were given according to the criteria presented in the method; *P < .05, **P < .01, ***P < .005 versus TNBS group.

The colons of the mice with TNBS-induced colitis showed evidence of mucosal congestion, erosion, loss of goblet cells, thickening of the colon wall and high level of leukocyte and polymorphonuclear (PMN) infiltration, while we observed improvement in the prognosis of the disease in groups treated with anthocyanins extract.  Figure 5(a) presents the representative photos of our section preparations, and Figure 5(b) illustrates the average histological score.

### 3.5. MPO Activity Measurement

**Figure 6:**
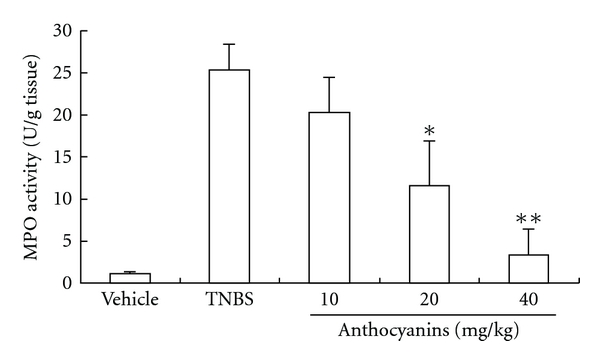
Effects of anthocyanins extract on MPO activity in TNBS-induced colitis in mice. Anthocyanins extract could significantly reduce the increased MPO activity in colon tissue induced by colitis. The data signify the average value of the tissues from all the surviving mice of each group, and the sample numbers of animal in each group were: 10, 3, 4, 5 and 7 for vehicle, TNBS control and mice treated with anthocyanins extract of 10, 20 and 40 mg kg^−1^, respectively; *P < .05, **P < .01, ***P < .005 versus TNBS group.

The MPO activity was used as an index of PMN infiltration. The MPO activity of vehicle, control and different doses of anthocyanins extract is shown in Figure 6. Mice from the control group demonstrated the highest MPO activity in colon, while 40 mg kg^−1^ of anthocyanins extract significantly inhibited the increase in MPO activity (*P* <  .01), and mice treated with 20 mg kg^−1^ of anthocyanins extract showed less MPO activity (*P* <  .05).

### 3.6. NO Release Measurement

**Figure 7:**
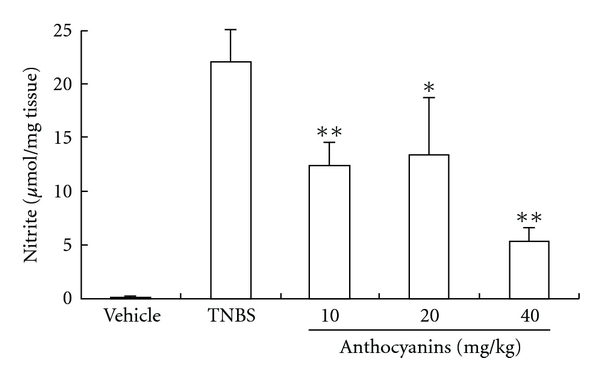
Effects of anthocyanins extract on the release of NO in TNBS-induced colitis in mice. All doses of anthocyanins extract significantly inhibited NO release in mice colon tissue; *P < .05, **P < .01, ***P < .005 versus TNBS group.

IBD and animal models of colitis were characterized by high levels of NO generation. As anthocyanins extract might act as a therapeutic agent for IBD through inhibition of NO production, we investigated the effects of anthocyanins extract in down-regulating the NO level in mice colon tissue. As shown in Figure 7, groups administered with 10 and 40 mg kg^−1^ anthocyanins extract significantly inhibited NO release induced by inflammation (*P* <  .01). In addition, 20 mg kg^−1^ of anthocyanins extract was also observed to reduce NO release (*P* <  .05), but the decrease was not in a dose-dependent manner.

### 3.7. Regulation of the Production of Both Pro-Inflammatory and Inhibitive Cytokines

**Figure 8:**
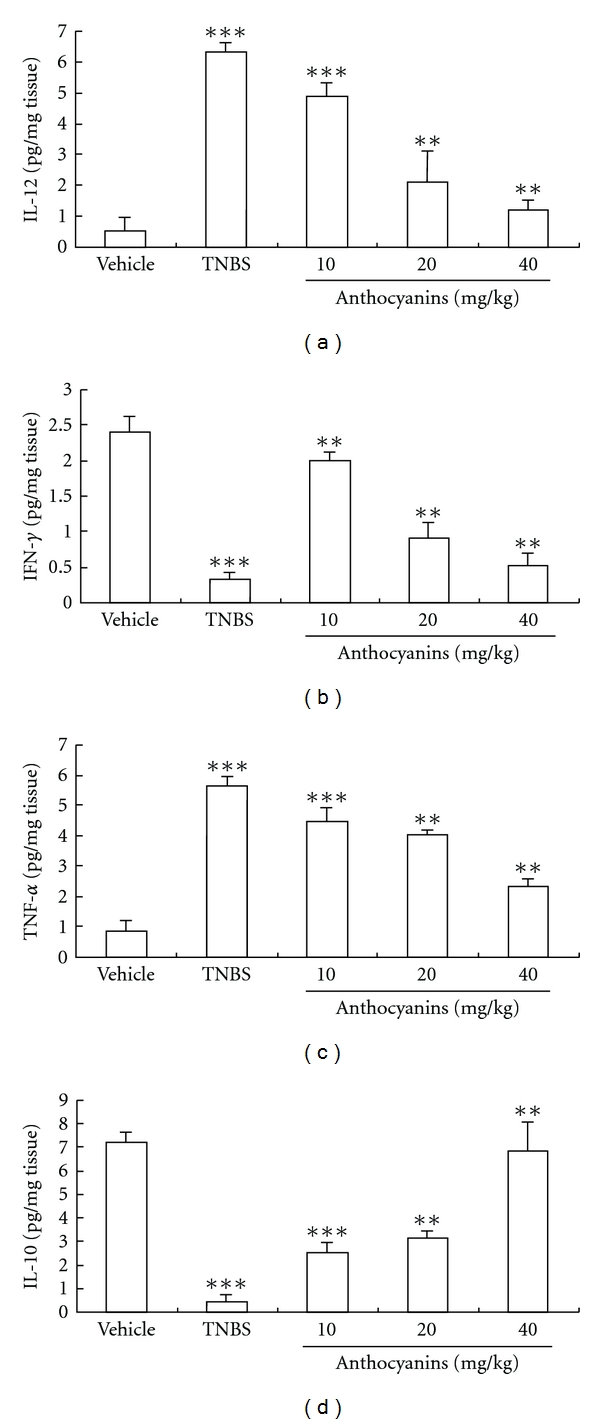
(a–d) Effects of anthocyanins extract on inflammatory cytokine production in mice with TNBS-induced colitis. All doses of anthocyanins extract significantly inhibited the production of IL-12 (a), IFN-*γ* (b) and TNF-*α* (c), as well as restored the product of IL-10 (d). *P < .05, **P < .01, ***P < .005 versus vehicle group.

As shown in Figure 8, all doses of anthocyanins extract from blueberries significantly reversed the increase in IL-12, TNF-*α* and IFN-*γ* levels, which were all high in TNBS control, and the production of IL-10 was up-regulated, which was inhibited in TNBS-induced colitis in mice. The disturbances in the production of these four main cytokines that play vital roles in the disease were balanced by anthocyanins extract in a dose-dependent manner.

## 4. Discussion

Although the etiology of IBD is still unclear, it is believed that altered immunological functions, resulting from the interplay between genetic susceptibility and certain environmental factors, can contribute significantly to the mucosal inflammation of the gastrointestinal tract. Moreover, it is often observed to lead to long-term and sometimes irreversible impairment of gastrointestinal structure and function [14]. When compared with the normal physiological situation, the abnormal pathological expressions of many pro-inflammatory cytokines, including TNF-*α*, IL-1*β* and -12, have been observed in IBD process. Nevertheless, the imbalance in the level of pro-inflammatory cytokines is involved in the maintenance of inflammatory response. In the animal model, intrarectal injection of TNBS is a well-characterized model of experimental colitis, which is comparable with the inflammatory processes present in IBD, and could demonstrate several important principles that are relevant to IBD. In this study, we used the TNBS-induced colitis model of mice to investigate the possible protective effect of anthocyanins extract on IBD. Another aim of the present study was to investigate some underlying mechanisms of the beneficial effects of anthocyanins extract from blueberries on experimental colitis in mice.

First, we found that anthocyanins extract from blueberries could directly improve the colitis symptoms in mice; all doses of anthocyanins extract prevented diarrhea as well as the resulting loss of body weight and mortality of the experimental mice. To further elucidate this protective effect, we investigated the effect of anthocyanins extract on colon lesion. It is well known that inflammation of colon could result in edema of large intestine, thus decreasing the length of the whole colon. In our experiment, the length of the colon from TNBS experimental mice reduced by ∼15–20% than that of the vehicle group. However, colons from mice that were administered with anthocyanins extract were longer than those from the TNBS group. Histological and macroscopic data confirmed the result that oral administration of anthocyanins extract from blueberries can alleviate the inflammation of the bowel. All these results demonstrate that anthocyanins extract from blueberries have protective effect on TNBS-induced colitis in mice.

Furthermore, anthocyanins are known to have vital physiological functions in plants owing to their antioxidant effect; MPO and NO are known sources of free radicals and can induce the reduction of ferritin (Fe^3+^) to free Fe^2+^, contributing to oxidative damage [15, 16]; both these components also play a very important role in the pathogenesis of IBD [17, 18]. Moreover, MPO is the enzyme produced mainly by PMN leukocytes and is associated with the degree of neutrophil infiltration in given tissues. Hence, we evaluated the effect of anthocyanins extract on colonic NO production and MPO activity. After 6 days of TNBS treatment, MPO activity of the TNBS group markedly increased to a level *∼*10 times higher than that found in the vehicle group. The increase in the MPO activity was significantly reduced by the administration of anthocyanins extract, and in the group administered with 40 mg kg^−1^ of anthocyanins extract, the MPO activity was suppressed almost to a basal level. As the MPO activity is considered to be a fundamental biochemical maker of neutrophil infiltration [19], this result suggests that anthocyanins extract exerted protective effects on IBD by reducing neutrophil infiltration into the colonic mucosa. In addition, the production of NO, another important mediator, was also down-regulated by the administration of different doses of anthocyanins extract. These results imply that some potential antioxidant natural products might have some important roles on IBD therapy.

**Figure 9:**
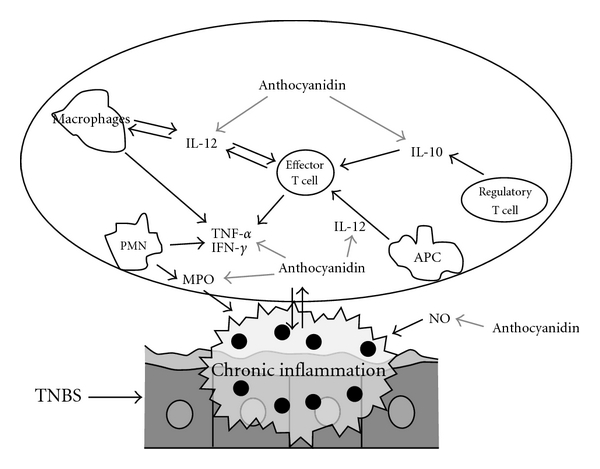
Main effects of the anthocyanins extract on the inflammatory intestinal process induced by TNBS in rats. Grey bold arrows indicate the inhibition in the inflammatory process.

Finally, TNBS-induced colitis in mice was much similar to IBD on human, which mainly resulted from Th1-mediated inflammation, and hence, we preliminarily investigated the regulation of anthocyanins extract on Th1-mediated cytokines that were involved in IBD. The results showed that anthocyanins extract from blueberries could significantly inhibit the increasing expression of IL-12, TNF-*α* and IFN-*γ*, as well as decrease the level of IL-10 (Figure 9). In the experimental treatment of IBD, evidence proved that the use of antibody specific for TNF-*α* was highly effective [20]. Similar effect was also observed when using IL-12 and -10 antibodies [21–24]. Our experimental results showed that anthocyanins extract from blueberries could significantly equilibrate the irregular expression of cytokines induced by colitis, and hence, we believe that the application of anthocyanins extract from blueberries could be an alternative to cytokines targeting therapy with lower cost and lesser side effect.

Thus, it can be concluded that intrarectal injection of TNBS in mice can cause severe colonic damage and inflammation. Treatment of mice with anthocyanins extract from blueberries significantly attenuated TNBS-induced inflammation of the murine colon. Our results suggest that the protective effect of anthocyanins extract may be linked to the re-equilibration of the irregular expression of cytokines induced by colitis. Therefore, we can presume that the high-dose intake of anthocyanins extract from blueberries (or blueberries) can have some beneficial effects on IBD.

## Funding

Opening fund of Jiangsu Province Key Laboratory for Plant Ex-Situ Conservation (KF08001). Grant from the Ministry of Agriculture of the People's Republic of China (nyhyzx07-028, 2006-G25).
